# Relationship between Sevoflurane Plasma Concentration, Clinical Variables and Bispectral Index Values during Cardiopulmonary Bypass

**DOI:** 10.1371/journal.pone.0134097

**Published:** 2015-08-27

**Authors:** Rainer Nitzschke, Joana Wilgusch, Jan Felix Kersten, Matthias Sebastian Goepfert

**Affiliations:** 1 Department of Anesthesiology, Center of Anesthesiology and Intensive Care Medicine, University Medical Center Hamburg-Eppendorf, Hamburg, Germany; 2 Department of Medical Biometry and Epidemiology, University Medical Center Hamburg-Eppendorf, Hamburg, Germany; University of Colorado Denver, UNITED STATES

## Abstract

**Background:**

Anesthetic administration is increasingly guided by electroencephalography (EEG)-based monitoring, such as the bispectral index (BIS). However, during cardiopulmonary bypass (CPB), factors other than the administered hypnotic agents may influence EEG signals, and their effects on BIS values are unknown.

**Methods:**

This report is a secondary analysis of data from a prospective, controlled interventional study comparing the effect of sevoflurane administration guided by BIS monitoring (group Sevo_BIS_) and constant administration of sevoflurane (group Sevo_1.8Vol%_) during CPB. Sevoflurane plasma concentration (SPC) was measured using gas chromatography. The relationships of BIS to SPC, CPB pump flow, arterial pressure, hematocrit, temperature, time on CPB, and patient characteristics were analysed.

**Results:**

No association was observed between BIS values and SPC in group Sevo_BIS_. In group Sevo_1.8Vol%_, a 40 μg ml^-1^ increase in SPC, which encompassed the entire range of observed values of the SPC in this analysis, was associated with a decrease of 3.6 (95% confidence interval (CI): 1.1–6.1) in BIS values (*p* = 0.005). Each increase in CPB time of 10 minutes was associated with an increase in BIS values of 0.25 (95%CI: 0.11–0.39, *p*<0.001). Path analysis revealed that the BIS values of Sevo_BIS_ patients were 5.3 (95%CI: 3.2–7.5) units higher than those of Sevo_1.8Vol%_ patients (*p*<0.001), which was the strongest effect on BIS values. Path analysis revealed a slope of 0.5 (95%CI: 0.3–0.7) BIS units per 1°C body temperature (p<0.001).

**Conclusion:**

BIS monitoring is insensitive to clinically relevant changes in SPC in individual patients during CPB.

## Introduction

The anesthetic requirement of a patient is nearly impossible to predict due to high between-patient and within-patient variabilities of patient needs and is particularly challenging for anesthesiologists during cardiopulmonary bypass (CPB) [1–3]. Therefore, many clinicians guide anesthetic delivery by monitoring the depth of sedation in cardiac surgery to prevent the under- or overdosing of anesthetics [3–6].

The most frequently used and validated monitor is the bispectral index (BIS, BIS-Monitor; Covidien, Boulder, CO, USA). Only limited portions of the proprietary algorithm by which physiological signals are translated into an index have been published [7–9].

Factors other than the hypnotic agents delivered during CPB may influence the EEG signal and therefore interfere with BIS monitoring. The CPB circuit’s pump flow and hemodilution may result in an uncertain cerebral oxygen supply, and hypothermia can potentially lead to suppression of the EEG; these alterations can lead to changes in BIS values [7, 10–12]. Other patient characteristics and comorbidities can also influence BIS values [2, 13, 14]. Because different causes of changes in BIS values have different therapeutic consequences, it is important for clinicians to understand factors that contribute to the origin of BIS indices during CPB.

We previously prospectively evaluated whether the administration of sevoflurane guided by BIS monitoring could reduce the sevoflurane plasma concentration (SPC) and intraoperative vasopressor doses in cardiac on-pump surgery [15]. Using these data, we conducted the present secondary analysis to investigate the relationships of changes in the SPC, CPB circuit pump flow, mean arterial blood pressure (MAP), hematocrit, patient temperature, duration of CPB, body mass index (BMI) and preoperative predicted operative mortality to BIS values during CPB. The following null hypothesis was tested: there are no relationships between these factors and BIS values during CPB.

## Materials and Methods

### Study design and patient population

This study was a secondary analysis of patients included in a prospective, controlled sequential 2-arm interventional study that was previously published [15]. Ethical approval for the study was provided by the regional review board of the Medical Council of Hamburg, Germany (reference number PV3623) and the trial was registered on ClinicalTrials.gov (ClinicalTrials.gov Identifier: NCT02515019). We report the results of a secondary analysis of the original study data that included only parts of the original patient population and only measurements during CPB.

Elective on-pump cardiac surgery patients who provided written informed consent were initially enrolled in the study between May and September 2011, and follow-up was completed for all patients. The inclusion criteria were an age of at least 18 years, elective on-pump cardiac surgery, an ASA status of III-IV and written informed consent. The exclusion criteria were a contraindication for the application of volatile anesthetics, an active infection with a body temperature > 38°C, and any history of neurological disease.

**Fig 1:**
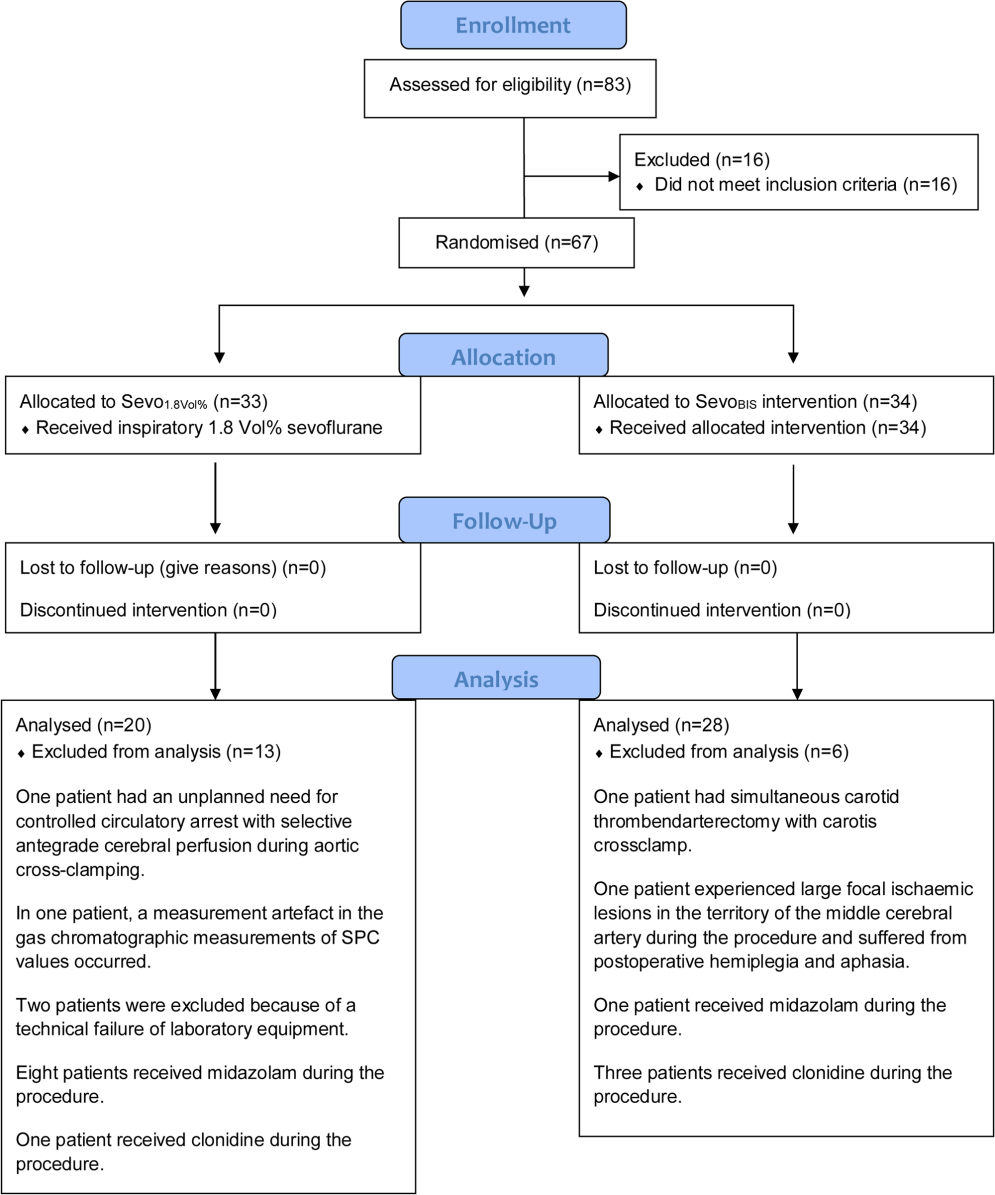
Patient flow chart showing information on participant losses and exclusions.

In total, 67 patients were initially enrolled in the prospective interventional study; of these, 19 patients were excluded from the post-hoc analysis for various reasons (Fig 1). Specifically, three patients were excluded from the BIS data analysis because of uncertainties in cerebral perfusion. In the first patient, a controlled circulatory arrest with selective antegrade cerebral perfusion was necessary during CPB. The second patient had a simultaneous carotid thromboendarterectomy with carotid cross-clamp during the study period. The third patient experienced large ischemic cerebral lesions during the procedure and awoke postoperatively with hemiplegia and aphasia. In addition, three patients were excluded from SPC analysis. Of these, an artefact was observed in the gas chromatographic measurements of the SPC for one patient, and gas chromatographic measurements were not performed on two patients due to a technical failure of laboratory equipment. Furthermore, we excluded 13 patients because they received additional bolus doses of midazolam or clonidine during the study period, which might have contributed to the sedative effect of sevoflurane and might have interfered with the BIS values. Subsequently, 48 patients with 386 measurements during CPB (162 in the control group and 224 in the intervention group) were included in this secondary analysis. For reproducibility, we present study data digitally as supporting information in the files “S1 Table Patient characteristics” and “S2 Table Intraoperative variables during CPB”.

### Study protocol

In 67 on-pump cardiac surgery patients, sevoflurane (Sevorane; Abbott, Wiesbaden, Germany) was administered via the respirator before CPB and via the oxygenator fresh-gas supply (Compactflo Evolution; Sorin Group, Milano, Italy) during CPB using an anesthetic vaporiser (Draeger Vapor Version 19.3 and Version 2000; Draeger, Luebeck, Germany). All patients received 7.5 mg of midazolam for premedication 60 minutes before the induction of anesthesia. Anesthesia was induced with 0.7 μg kg^-1^ intravenous sufentanil, followed by 1.3–2.0 mg kg^-1^ propofol and 0.1 mg kg^-1^ pancuronium bromide. Analgesia was maintained with a constant administration of 0.7 μg kg^-1^ h^-1^ sufentanil, and muscle relaxation was maintained with bolus applications of pancuronium bromide 0.05 mg kg^-1^ every 120 minutes. An adequate depth of sedation was maintained according to the study protocol. The details of anesthetic management, the CPB technique and the surgical procedure were previously described [15].

Thirty-four patients were allocated to an intervention group (Sevo_BIS_) with BIS-guided administration, in which sevoflurane was titrated to target BIS values between 40 and 60. Thirty-three patients were allocated to a control group (Sevo_1.8Vol%_) in which inspiratory 1.8 Vol% sevoflurane was administered continuously via the respirator before CPB and in the oxygenator fresh-gas supply during CPB. The inspiratory concentration of 1.8 Vol% sevoflurane resulted in an average of one age-adjusted minimum alveolar concentration (aaMAC).

Thirty-second moving average BIS values were sampled digitally at 1-minute intervals (BIS Vista Version 3.0) and stored electronically. During the study period, we collected blood samples for gas chromatographic analysis of the SPC from the radial artery catheter at predefined measurement time points. A gas chromatography-flame ionisation detector (Clarus 500; PerkinElmer, Shelton, UK) with an automatic sampler for headspace analysis (TurboMatrix; HS 40, PerkinElmer) was used to measure the SPC; the details of the laboratory measurements were previously published [16]. A research fellow who was not involved in patient care conducted the data collection during the study period, the blood sampling and the gas chromatographic analysis.

In the original trial, the primary endpoint was the effect of BIS-guided administration of sevoflurane on the amount of SPC during CPB and the intraoperative required cumulative dose of norepinephrine. The patients were followed up postoperatively with assessments of blood lactate concentration, duration of mechanical ventilation, acute kidney injury, intensive care unit length of stay and an interview about intraoperative awareness. In brief, patients in the BIS-guided group had a lower SPC and higher MAP and required significantly less norepinephrine during on-pump cardiac surgery compared with the control group.

The present secondary analysis assessed the association between the SPC, individual characteristics, clinical variables and parallel recorded BIS values during the time period on CPB.

### Statistical analysis

Categorical data are presented as counts (percentages), whereas continuous data are expressed as means with 95% confidence intervals (CIs) or as means with standard deviations (SDs). The data were checked for normal distribution before analysis.

Pearson’s correlation was used to measure the bivariate association between SPC and BIS values in the study groups.

The effects of the independent variables SPC, CPB circuit pump flow, MAP, hematocrit, patient temperature, time on CPB, BMI and the preoperative predicted operative mortality (EuroSCORE II) on BIS values during CPB were estimated by fitting a hierarchical analysis of covariance [ANCOVA] model (linear mixed random intercept model) to the data, with first-order autocorrelation between consecutive time points and assuming homoscedasticity between measurements. The model takes into account repeated measurements within the same patient and quantifies the relationships between the covariates and the dependent variable. The marginal means estimated by the model are reported.

In addition, we performed a path analysis to describe the interdependencies between the exogenous variables and to estimate the comparative strength of their effects on the dependent variable [17]. Competing path models were evaluated, and global goodness of fit was assessed via the likelihood ratio test, the root mean square error of approximation (RMSEA) and the comparative fit index (CFI). We report standardised path coefficients (β), which represent partial regression coefficients and enable comparisons of the effects of the independent variables [17].

Two-tailed p-values <0.05 were considered significant. All statistical analyses were performed using the statistical software packages SPSS version 22 (IBM SPSS Statistics Inc., Chicago, IL, USA), R statistical environment 3.1.1 (R Foundation for Statistical Computing, Vienna, Austria) and Mplus Version 5.1 (Muthen and Muthen, Los Angeles, CA, USA).

## Results

### Bivariate association between SPC and BIS

The duration of CPB ranged from 58 to 266 min. During CPB, some values were missed: 1.0% (4/386) of the SPC measurements, 0.3% (1/386) of blood gas analysis and 0.8% (3/386) of patient temperature measurements. The patient characteristics and intraoperative variables of patients included in this secondary analysis are presented in Table 1. In this study population, the mean SPC achieved with the administration of sevoflurane via the oxygenator during CPB was 22.2 (±9.8) μg ml^-1^ in the Sevo_BIS_ group and 46.1 (±7.4) μg ml^-1^ in the Sevo_1.8Vol%_ group. The mean BIS values were 42.5 (±4.7) in the Sevo_BIS_ group and 37.2 (±6.9) in the Sevo_1.8Vol%_ group.

**Table 1:** Characteristics of the study population.

**Demographics**	
Number of patients	48
Age (yrs)	66.2 (±9.8)
Female gender	19 (40%)
Body mass index (kg/m^2^)	28.8 (±5.5)
EuroSCORE II	1.97 (±1.7)
Preoperative LV-Fx:	
Normal	38 (79%)
Mildly impaired	5 (10%)
Moderately impaired	4 (8%)
Severely impaired	1 (2%)
**Intraoperative variables during CPB (4–10 measurements per patient)**	
CPB duration (min)	131 (±47)
Aortic cross-clamp time (min)	85 (±31)
Pump flow (l * min^-1^ * m^2^)	2.53 (±0.30)
Body temperature (°C)	33.8 (±2.3)
Mean arterial blood pressure (mmHg)	63 (±8)
Hematocrit (%)	27.1 (±4.6)
Arterial CO_2_ partial pressure (mmHg)	42.2 (±5.2)
Blood lactate concentration (mmol * l^-1^)	1.2 (±0.8)
Blood glucose concentration (mmol * l^-1^)	9.5 (±3.1)
Patients receiving erythrocyte concentrate transfusion	17 (35%)
**Surgical procedure**	
Exclusively coronary bypass surgery	25 (52%)
Aortic valve replacement or open heart surgery	17 (35%)
Combined simultaneous aortic valve replacement and coronary bypass surgery	6 (13%)

The data are presented as means (SD) for continuous data and numbers (%) for categorical data; LV-Fx = Left ventricular function; CPB = Cardiopulmonary bypass; EuroSCORE II = European System for Cardiac Operative Risk Evaluation II.

**Fig 2:**
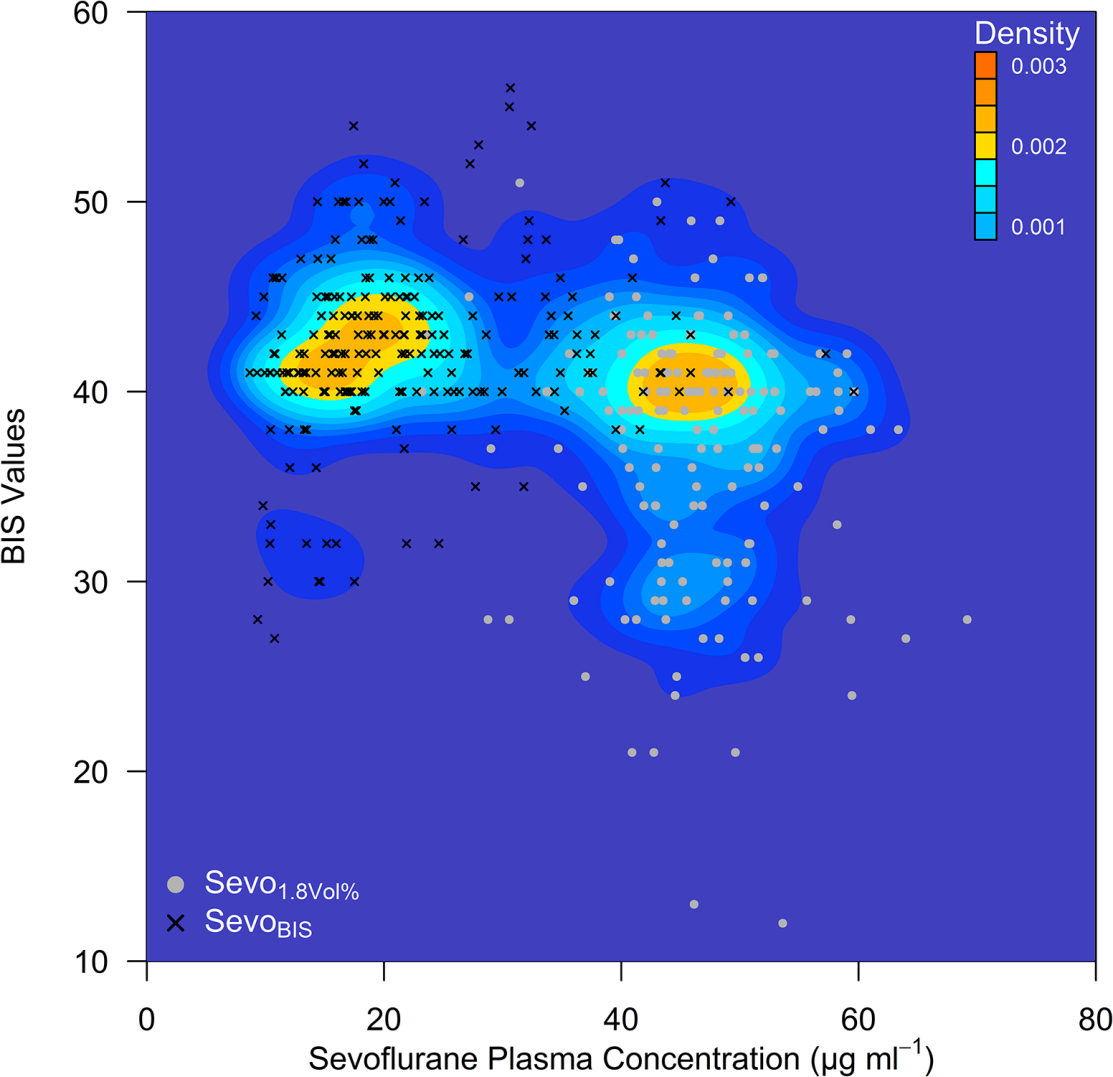
Contour plot showing the density of BIS values and the Sevoflurane Plasma Concentration. Sevoflurane Plasma Concentration measured in blood samples by gas chromatography during CPB. The plot is based on 159 measurements in the control group (Sevo_1.8Vol%_) and 223 measurements in the intervention group (Sevo_BIS_) from 48 (20/28) individuals. When the data were analysed using a bivariate method, there was a minimal significant positive correlation between BIS values and the SPC (Pearson’s r 0.172, 95% CI: 0.042 to 0.297, *p* = 0.010) in the intervention group, Sevo_BIS_. In the bivariate analysis, there was no significant correlation between the BIS values and the SPC (Pearson r -0.119, 95% CI: -0.269 to 0.038, *p* = 0.136) in the Sevo_1.8Vol%_ group.

The bivariate distribution of BIS values during CPB in comparison to the SPC for the Sevo_BIS_ group and the Sevo_1.8Vol%_ group is presented in a contour plot (Fig 2). Most BIS values were between 35 and 45 in both groups.

### Linear Mixed-Effects Model Results

The results of the multivariate regression analysis are presented in Table 2. The mixed-effect model analysis revealed that in the Sevo_1.8Vol%_ group, an increase in the SPC of 10 μg ml^-1^ caused an estimated decrease in the BIS values of 0.9. Thus, in the Sevo_1.8Vol%_ group, an increase in the SPC of 40 μg ml^-1^, which encompasses the entire range of the SPC in the study population, would only result in a decrease of 3.6 in BIS values. In the Sevo_BIS_ group, there was no significant association between BIS values and the SPC. Multivariate regression analysis also indicated that every 10-minute increase in the time on CPB caused an increase of approximately 0.25 in BIS values in both groups. There were no significant associations of BIS values with patient temperature, mean arterial blood pressure, hematocrit, CPB pump flow, BMI and the patient’s EuroSCORE II. Interdependencies also revealed no significant correlations. The covariance structure indicated that the intraclass correlation coefficient was 41% (*p*<0.001) in the study population, i.e.; 41% of the variance in the BIS values was explained by the individual subjects.

**Table 2:** Regression coefficients as assessed using the mixed-effects model describing the relationship between BIS values as the response variable and different independent variables during CPB.

Independent Variable	Estimate	95% CI	SE	*p* value
SPC group Sevo_1.8Vol%_ (μg ml^-1^)	- 0.090	-0.153 to -0.03	0.028	0.005
SPC group Sevo_BIS_ (μg ml^-1^)	0.037	-0.057 to 0.131	0.05	0.441
Pump flow (l min^-1^ m^-2^)	0.966	-0.852 to 2.785	0.92	0.296
MAP (mmHg)	-0.027	-0.097 to 0.043	0.04	0.452
Hematocrit (%)	-0.005	-0.142 to 0.132	0.07	0.944
Temperature (°C)	0.079	-0.183 to 0.341	0.13	0.554
Time on CPB (min)	0.025	0.011 to 0.039	0.01	<0.001
BMI (kg m^-2^)	-0.175	-0.403 to 0.054	0.11	0.132
EuroSCORE II	-0.366	-1.114 to 0.383	0.37	0.331

SE, Standard error; CI, Confidence interval; SPC, Sevoflurane plasma concentration; MAP, Mean arterial blood pressure; CPB, Cardiopulmonary bypass; BMI, Body mass index; EuroSCORE II, European System for Cardiac Operative Risk Evaluation II.

### Results of the Path Analysis

**Fig 3:**
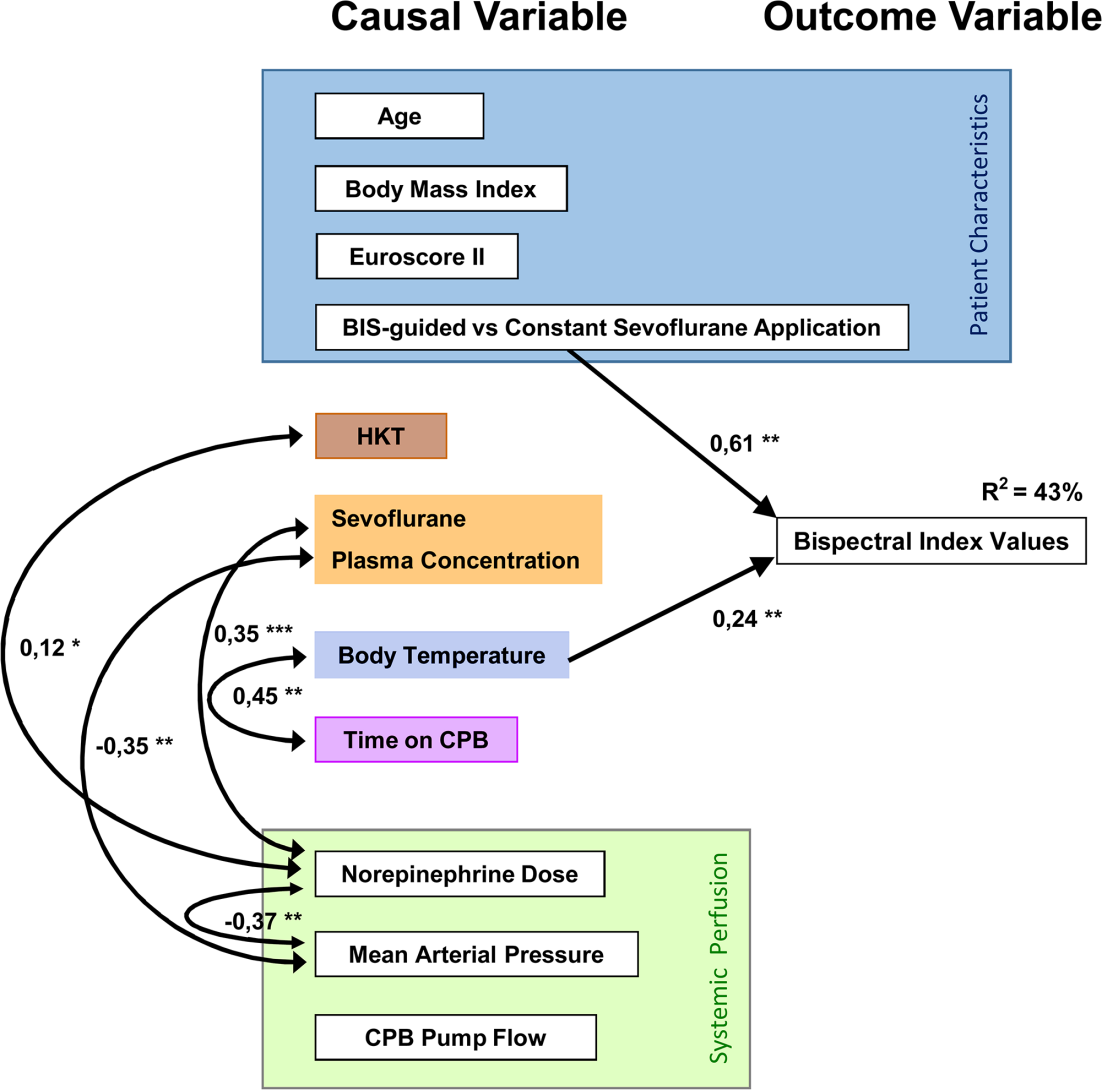
Final path model. The path model was calculated to estimate the relationships between BIS values as the only endogenous variable and different exogenous variables during CPB. The single-headed arrows represent the regression coefficients; the arrows lead from the explanatory (causal) variable to the outcome variable (effect). The double-headed arrows represent correlations. The figures under the arrowed lines are standardised path coefficients, which must be interpreted according to the product-moment correlation as follows: >.1 = weak effect; >.3 = moderate effect; >.5 = strong effect. The model variables accounted for 43% of the variance of the BIS values. (* = *p* < 0.01; ** = *p* < 0.001). EuroSCORE II, European System for Cardiac Operative Risk Evaluation II; HKT, Hematocrit; CPB, Cardiopulmonary bypass.

The path analysis was calculated using a series of multiple regression analyses based on the hypothesised model, and the results are presented in Table 3 and in a graphical path model (Fig 3). The final model had an acceptable fit with χ^2^ test = 48.62 (df = 15, *p*<0.001), RMSEA = 0.068 and CFI = 0.882. Path analysis demonstrated that BIS values were 5.3 (95% CI: 3.2 to 7.5) units higher in Sevo_BIS_ patients compared to Sevo_1.8Vol%_ patients (*p*<0.001). In addition, the analysis revealed a slope of 0.5 (95% CI: 0.3 to 0.7) BIS units per 1°C body temperature (*p*<0.001). The allocation of a patient to the group Sevo_BIS_ or group Sevo_1.8Vol%_ had a strong effect (β = 0.611, *p*<0.001) on BIS values, whereas body temperature had a moderate effect (β = 0.241, *p<*0.001) on BIS values during CPB. The path model did not reveal any other causal effects between the variables in the model and BIS values. R^2^ was 0.058 (*p* = 0.021) for the between-patient variable allocation to the Sevo_BIS_ or Sevo_1.8Vol%_ groups, whereas the R^2^ was 0.373 (*p* = 0.002) for the within-patient variable body temperature. Thus, the model variables accounted for 43% of the variance in the BIS values.

**Table 3:** Causal effects on BIS values and correlations among exogenous variables with standardized path coefficients based on the final path analysis.

Causal Effect	Standardised Path Coefficient β	95% CI	SE	*p* value
Allocation to BIS-guided or constant sevoflurane application → BIS values	0.611	0.414 to 0.807	0.100	<0.001
Body temperature → BIS values	0.241	0.139 to 0.344	0.052	<0.001
**Correlation**	**r**			
Norepinephrine dose ↔ MAP	-0.374	-0.459 to -0.288	0.043	<0.001
Norepinephrine dose ↔ SPC	0.350	0.262 to 0.438	0.045	<0.001
Norepinephrine dose ↔ HKT	0.118	0.030 to 0.207	0.045	0.009
Body temperature ↔ Time on CPB	0.449	0.368 to 0.529	0.041	<0.001
MAP ↔ SPC	-0.346	-0.434 to -0.258	0.045	<0.001

CI, Confidence interval; SE, Standard error; BIS, Bispectral index; MAP, Mean arterial blood pressure; SPC, Sevoflurane plasma concentration; HKT, Hematocrit; CPB, Cardiopulmonary bypass; Interpretation of standardized path coefficients β according to product-moment correlation: >.1 = weak effect; >.3 = moderate effect; >.5 = strong effect.

## Discussion

This analysis identified only a limited statistical relationship between BIS values and the SPC during CPB. Despite significant differences in SPCs among patients, most BIS values were approximately 40.

The multivariate regression analysis did not reveal any systematic association between the SPC and BIS values in the BIS-guided group. The lack of an association could be due to the feedback effect of the intervention itself. The physicians reduced sevoflurane administration in patients with lower BIS values and increased sevoflurane administration when patients displayed higher values. In the control group in which sevoflurane was administered continuously, there was a statistically significant but clinically irrelevant relationship between BIS values and the SPC. The association of BIS values for subjects allocated to either the BIS-guided or the control group with the difference in the level of the SPC between the two groups was the strongest relationship identified in the multivariate analysis. The present analysis determined that the time on CPB and the patient’s temperature had significant but clinically irrelevant positive correlations with BIS values. The results suggest that the measured BIS values are insensitive to relevant changes of the SPC and hemodynamic variables during CPB.

The manufacturer of the BIS monitoring devices associates deep general anesthesia with a low index value. However, there are data suggesting that low BIS values in cardiac surgery may be a consequence of relative anesthetic overdose as well as low organ perfusion and high patient morbidity [2, 6, 13, 14, 18].

BIS monitoring might prevent inappropriately light depths of sedation with BIS values above 60 and limit the risk of intraoperative awareness [19, 20]. In addition, BIS monitoring prevents excessive doses of anesthetics with avoidable cardiocirculatory depression by targeting BIS values above 40 [2, 15, 20]. Several publications have reported a plateau of intraoperative BIS values at approximately 40 with the use of isoflurane, sevoflurane, desflurane and propofol in non-cardiac surgery, in which the anesthetic concentration can be increased or decreased without an effect on the BIS value [21–24]. Accordingly, the authors of a large trial in which a total of more than three million intraoperative data points from 1,100 patients were retrospectively analysed observed only a weak association between the BIS during the maintenance phase of general anesthesia and end-tidal volatile anesthetic gas concentrations (ETAC). They also reported a plateau at which BIS values remained in the low 40s over the ETAC range from 0.42 to 1.51 aaMAC. They calculated that for every aaMAC increase in ETAC, the BIS value would decrease by an estimated 8 units [25]. In another analysis of a large pool of data published by the same group of authors, increasing aaMAC by 0.1 resulted in a calculated average decrease in BIS values of 1.8 in patients without a history of intraoperative awareness [26]. This low correlation between BIS values and administered anesthetics can be interpreted to indicate that BIS monitoring does not reflect changes in the depth of anesthesia apart from whether the patient is unconscious or awake. However, the published plateau of BIS values might indicate that spontaneous electrical brain function does not change during deep anesthesia over a wide range of anesthetic concentrations [20]. The present analysis does not contribute to the clarification of this issue because parallel electroencephalographic recordings were not analysed.

Despite this low association between BIS values and the measured anesthetic concentration within the plateau in the low 40s, avoiding BIS values below 40 helps to avoid excessive doses of anesthetics and to decrease vasopressor administration compared with routine care during on-pump cardiac surgery [15].

Factors other than the delivered hypnotic agents may decrease the frequency of the spontaneous EEG signal during CPB. Hypothermia can lead to EEG suppression and might consequently interfere with BIS monitoring [7, 10]. In a post-hoc analysis of the B-Unaware trial, the duration of periods with BIS values less than 45 was associated with mild hypothermia of 32°C to 34°C during cardiopulmonary bypass [13]. In prospective studies, the BIS was estimated to decline between one and two units per 1°C decrease in body temperature during CPB with mild hypothermia [11, 12, 27, 28]. BIS values decreased more rapidly during deep hypothermia than during mild hypothermia, and BIS values declined to zero after the body temperature reached 17°C [29]. However, the published data cannot definitively address whether the lower BIS values observed during hypothermic CPB are a result of brain cooling, an inaccuracy of the BIS algorithm at lower temperatures, or an increased plasma concentration of anesthetics because of changes in pharmacodynamics [12, 27, 30]. The mean body temperature in our study was 33.8 ± 2.3°C. We therefore assume that the lack of a significant relationship between temperature and BIS values in our data occurred because in this temperature range, BIS indices are only slightly affected by hypothermia.

Although the hypothesis is tempting, it is unclear if BIS values are influenced by cerebral perfusion. The durations of periods with BIS values less than 45 seem to be associated with intraoperative MAPs less than 55 mmHg [13]. No data have been published to date on the association between CPB pump flow and BIS values.

The present study observed a statistically significant positive association between BIS values and CPB duration that cannot be interpreted as clinically relevant. In principle, the present analysis observed no clinically significant association between the duration of CPB and BIS values, while previous studies have demonstrated a tendency toward an increase in the sensitivity of the brain to anesthetics during and after CPB, resulting in a decreased aaMAC, an increase in the burst suppression ratio and decreased BIS values [13, 31–33].

There is some evidence that low BIS values might occur in vulnerable patients with underlying frailty, who are more sensitive to anesthetic exposure [13, 14, 25]. Patients with ASA physical status greater than 3 or with poor left ventricular function had lower BIS values than other patients [13, 25]. Our data could not indicate a relationship between the predicted operative mortality assessed with the EuroSCORE II and BIS values during CPB in our patients.

The present study has some limitations. First, this secondary analysis only analysed the relationships using data collected during CPB and does not permit the generalisation of the results to all patients during general anesthesia. Second, the sample size might be insufficient to derive significant additional correlative information regarding patient factors that influence BIS values. Third, path analysis is a statistical method that uses correlational data to identify the various causal relations underlying a particular outcome. As a limitation, the relationships cannot be tested for directionality, and the models themselves cannot prove causation. However, path models evaluate which hypothesised causal model best fits the pattern of correlations within the data set [17]. Furthermore, in this study, while BIS recordings during the operation were accompanied by raw EEG recordings, these EEG signals were not digitally stored, and parallel conventional multichannel EEG recordings were not conducted. Therefore, we could only correlate the clinical findings with BIS values without further assessing the correlation of the underlying raw EEG signals with BIS values. Interestingly, there are data indicating that in the late CPB period, the raw EEG changes from a slow, synchronous, large-wave rhythm to an EEG rhythm accompanied by disproportional de-synchronous activity, even though the BIS values recorded in parallel remained unchanged [34]. Future studies should be conducted with BIS monitoring and parallel EEG recordings to clarify if EEG changes occur during the plateau phase of BIS values or if electrical brain function does not change during relatively deep anesthesia.

## Conclusion

In conclusion, the study findings demonstrate that apart from the association of BIS values with the allocation of the patient to either the BIS-guided or the control group, BIS monitoring is insensitive to clinically relevant changes of the SPC within individual patients during CPB. In addition, there is no relevant relationship between BIS values and changes in hemodynamic parameters during CPB.

## Supporting Information

S1 Table Patient characteristics. (XLSX)

S2 Table Intraoperative variables during CPB. (XLSX)
